# Azido­(η^5^-penta­methyl­cyclo­penta­dien­yl)[2-(pyridin-2-yl)phen­yl]iridium(III)

**DOI:** 10.1107/S1600536813026159

**Published:** 2013-09-28

**Authors:** Keita Ariyoshi, Takayoshi Suzuki

**Affiliations:** a Department of Chemistry, Faculty of Science, Okayama University, Tsushimanaka 3-1-1, Okayama, 700-8530, Japan

## Abstract

In the title compound, [Ir(C_10_H_15_)(C_11_H_8_N)(N_3_)], the Ir^III^ ion is coordinated by three anionic ligands, namely, penta­methyl­cyclo­penta­dienyl (Cp*^−^), 2-(pyridin-2-yl)phenyl (ppy^−^) and azide (N_3_^−^), and adopts a three-legged piano-stool geometry The coordination mode of N_3_^−^ is typical for Cp*Ir^III^–N_3_ complexes, with an Ir—N(N_3_) bond length of 2.125 (2) Å and an Ir—N=N bond angle of 116.5 (2)°. The N_3_^−^ ligand is almost linear [N=N=N = 176.0 (3)°], and the N=N bond length between the central and coordinating N atom and that between the central and non-coordinating terminal N atom are 1.194 (3) and 1.157 (3) Å, respectively. For the ppy^−^ ligand, the Ir—C and Ir—N bond lengths are 2.066 (3) and 2.079 (3) Å, respectively, which are rather close to each other, compared to the related Ir^III^– or Rh^III^–ppy complexes. The Ir—C(Cp*) bond lengths vary in the range 2.163 (2)–2.232 (2) Å, indicating a strong *trans* influence of the cyclo­metallated C-donor atom of the ppy^−^ ligand.

## Related literature

For crystallographic analyses of [Cp*Ir^III^(N_3_)(L–L′)] (L–L′ = bidentate chelate ligands; *e.g.*, bpy, 2-Spy, *etc*.) complexes, see: Suzuki *et al.* (2009 ▶); Suzuki (2005 ▶). For crystallographic analyses of mononuclear [Cp*Ir(ppy)X] complexes (X = Cl, I, MeCN_4_, *etc.*), see: Boutadla *et al.* (2009 ▶); Park-Gehrke *et al.* (2009 ▶); Takayama *et al.* (2013 ▶). For photochemistry of [Cp*Ir^III^(N_3_)(L–L′)] complexes, see: Sekioka *et al.* (2005 ▶); Kotera *et al.* (2008 ▶). 
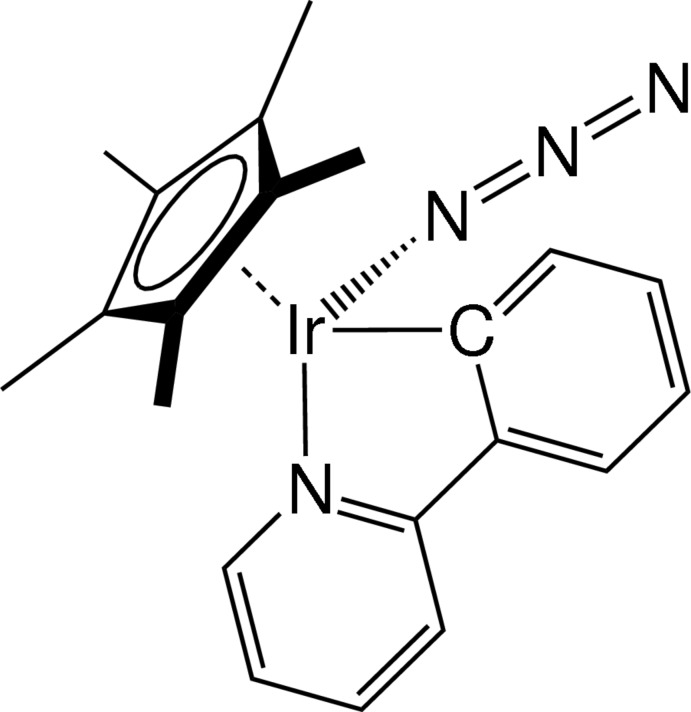


## Experimental

### 

#### Crystal data

[Ir(C_10_H_15_)(C_11_H_8_N)(N_3_)]
*M* *_r_* = 523.63
Monoclinic, 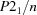

*a* = 15.4821 (18) Å
*b* = 7.3938 (9) Å
*c* = 15.7137 (18) Å
β = 91.477 (4)°
*V* = 1798.2 (4) Å^3^
*Z* = 4
Mo *K*α radiation
μ = 7.44 mm^−1^
*T* = 193 K
0.30 × 0.30 × 0.20 mm

#### Data collection

Rigaku R-AXIS RAPIDII diffractometer
Absorption correction: numerical (*NUMABS*; Rigaku, 1999 ▶) *T*_min_ = 0.103, *T*_max_ = 0.225
27640 measured reflections
4115 independent reflections
4022 reflections with *I* > 2σ(*I*)
*R*_int_ = 0.045

#### Refinement

*R*[*F* ^2^ > 2σ(*F* ^2^)] = 0.017
*wR*(*F* ^2^) = 0.042
*S* = 1.13
4115 reflections
240 parameters
H-atom parameters constrained
Δρ_max_ = 1.61 e Å^−3^
Δρ_min_ = −0.65 e Å^−3^

### 

Data collection: *RAPID-AUTO* (Rigaku, 2006 ▶); cell refinement: *RAPID-AUTO*; data reduction: *RAPID-AUTO*; program(s) used to solve structure: *DIRDIF99-PATTY* (Beurskens *et al.*, 1999 ▶); program(s) used to refine structure: *SHELXL2013* (Sheldrick, 2008 ▶); molecular graphics: *ORTEP-3 for Windows* (Farrugia, 2012 ▶); software used to prepare material for publication: *SHELXL2013*.

## Supplementary Material

Crystal structure: contains datablock(s) I, global. DOI: 10.1107/S1600536813026159/is5306sup1.cif

Structure factors: contains datablock(s) I. DOI: 10.1107/S1600536813026159/is5306Isup2.hkl

Additional supplementary materials:  crystallographic information; 3D view; checkCIF report

## Figures and Tables

**Fig. 1.**
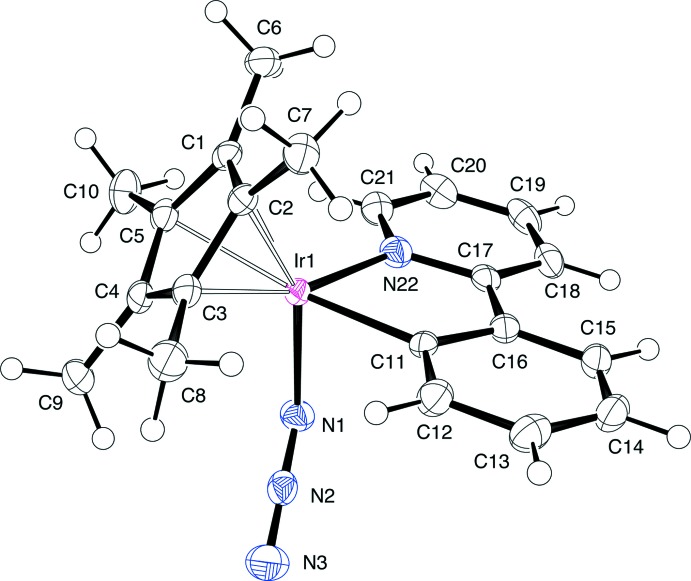
The molecular structure of the title compound with atom-numbering scheme. Displacement ellipsoids are drawn at 50% probability level.

## supplementary crystallographic information

### 1. Comment

In previous studies we have prepared a number of iridium(III) azido complexes, [Cp*Ir^III^(N_3_)(L–L')] (Cp* = pentamethylcyclopentadienyl, L–L' = various kinds of bidentate chelate ligands), and investigated their structures and photochemical reactivities. Among them, complexes of [Cp*Ir(N_3_)(Me_2_dtc)] (Me_2_dtc^-^ = *N*,*N*-dimethyldithiocarbamate) and [Cp*Ir(N_3_)(2-Spy)] (2-Spy^-^ = 2-pyridinethiolate) afforded interesting photolysis products with two-legged piano-stool structures, [Cp*Ir{SC(NMe_2_)SN}] and [Cp*Ir(1-N-2Spy)], respectively, by insertion of a N-atom originating from the coordinated azido ligand, into the Ir–S and Ir–N(py) bonds, respectively (Sekioka *et al.*, 2005). In contrast, photolysis of the related complexes with an N—N, N—P or P—P type four-membered chelate ligand (*i.e.*, 1,8-naphthyridine, 2-diphenylphosphinopyridine or bis(dimethylphosphino)methane) gave a complicated mixture of uncharacterized products, due probably to reductive elimination of the coordinated azide (Suzuki *et al.*, 2009). In the case of [Cp*Ir(N_3_)(bpy)]PF_6_ (bpy = 2,2'-bipyridine), photolysis in acetonitrile produced a 5-methyltetrazolato complex, [Cp*Ir(N_3_)(MeCN_4_)]^+^, which was confirmed by ^1^H NMR spectroscopy (Kotera *et al.*, 2008). In addition, the X-ray structural analysis of the bpy complex, [Cp*Ir^III^(N_3_)(bpy)]PF_6_, revealed some structural characteristics different from those of the other [Cp*Ir(N_3_)(L–L')] complexes (Suzuki, 2005; Suzuki *et al.*, 2009). For instance, the Ir—N(N_3_) bond in the bpy complex [2.230 (6) Å] was longer by *ca* 0.1 Å than the typical Ir—N(N_3_) bond lengths in the other [Cp*Ir(N_3_)(L–L')] complexes. Triatomic unit of N_3_^-^ was almost linear as usual, but the N—N bond length between the central and coordinated N atoms was unusually longer by *ca* 0.25 Å than that between the central and non-coordinated terminal N atoms. In this study, we have prepared and characterized the analogous Cp*Ir^III^(N_3_) complex with a structurally similar but an anionic 2-(pyridin-2-yl)phenyl (ppy^-^) ligand, [Cp*Ir(N_3_)(ppy)].

The title compound crystallized in a monoclinic space group *P*2_1_/*n* with *Z* = 4. The Ir^III^ ion was coordinated by three anionic ligands, Cp*^-^, ppy^-^ and N_3_^-^, and it took a three-legged piano-stool structure. The ppy^-^ ligand formed a planar chelate, having the Ir1—C11 bond of 2.066 (2) Å and the Ir1—N22 bond of 2.079 (2) Å. It is noted that the difference between the Ir—C and Ir—N bond lengths is not so large (0.013 Å), compared to the typical Ir^III^ or Rh^III^ (*M*^III^)–ppy complexes, where the M—C bond is significantly shorter than the M—N bond (Takayama *et al.*, 2013). In some cases of Ir^III^–ppy complexes with a simple halide, similarly small differences in the Ir—C and Ir—N bonds were also reported; for example, 0.016 Å in [Cp*IrCl(ppy)] (Boutadla *et al.*, 2009) and 0.029 Å in [Cp*IrI(ppy)] (Park-Gehrke *et al.*, 2009). These small differences may be due to a partial configurational disorder of the ppy coordination.

The Ir1—N1 coordination bond length is 2.125 (2) Å and the Ir1—N1—N2 bond angle is 116.5 (2)°, while the triatomic azide moiety is almost linear: N1—N2—N3 176.0 (3)°. The N1—N2 and N2—N3 bond lengths are 1.194 (3) and 1.157 (3) Å, respectively. These structural parameters are typical for [Cp*Ir^III^(N_3_)(L–L')] complexes (Suzuki *et al.*, 2009), except for [Cp*Ir(N_3_)(bpy)]PF_6_ (Suzuki, 2005).

The five Ir1—Cn(Cp*) bond lengths are 2.175 (2), 2.163 (2), 2.201 (2), 2.230 (2) and 2.232 (2) Å for n = 1–5, respectively (Table 1). Two relatively long (to C4 and C5) bonds are approximately *trans* to the C-donor atom of ppy^-^ ligand. A similar elongation of the Ir—C bonds are also observed in the other mononuclear [Cp*Ir^III^(ppy)*X*] complexes (Park-Gehrke *et al.*, 2009; Takayama *et al.*, 2013), indicating a strong *trans* influence of the cyclometalated C-donor. The Ir1—C3 bond, which is *trans* to the N-donor of ppy^-^, is a little longer than the other two; this may indicate a partial configurational disorder of the N– and C-donor of ppy^-^ ligand in the Cp*Ir^III^(ppy) complexes.

In the crystal structure there are no solvent molecules of crystallization. Further, any characteristic intermolecular interaction is not observed in this crystal.

When UV light was irradiated to an acetonitrile solution of this complex, a tetrazolato complex, [Cp*Ir(ppy)(MeCN_4_)] (Takayama *et al.*, 2013) was formed, which was confirmed by ^1^H NMR spectroscopy.

### 2. Experimental

A methanol solution (20 cm^3^) of NaN_3_ (377 mg, 5.18 mmol) was added with stirring to an orange solution of [Cp*IrCl(ppy)] (260 mg, 0.503 mmol) in a 1:1 mixture of methanol and dichloromethane (15 cm^3^). The color of mixture turned to yellow immediately, and yellow precipitate was formed. After stirring at room temperature for 5 h, the reaction mixture was evaporated to dryness under reduced pressure. The residue was extracted with dichloromethane (50 cm^3^), and the filtered extract was concentrated under reduced pressure. Diethyl ether vapor was diffused into the concentrate in a closed vessel, affording orange needle crystals. Yield: 208 mg (79%). Anal. Found: C 48.01, H 4.16, N 10.56%. Calcd for C_23_H_23_IrN_4_: C 48.16, H 4.43, N 10.70%. ^1^H NMR (400 MHz, 21 °C, CD_3_CN): δ 1.68 (s, Cp*, 15H), 7.09 (t, *J* = 7.3 Hz, ppy, 1H), 7.18–7.26 (m, ppy, 2H), 7.77–7.87 (m, ppy, 3H), 7.98 (d, *J* = 8.1 Hz, ppy, 1H) and 8.71 (d, *J* = 5.4 Hz, ppy, 1H). IR (KBr disc): ν(N_3_) = 2027 cm^-1^.

### 3. Refinement

All H atoms were positioned geometrically and refined using a riding model with C—H = 0.95 (for aromatic) or 0.98 Å (for methyl) and with *U*_iso_(H) = 1.2 (for aromatic) or 1.5 (for methyl) *U*_eq_(C).

### Crystal data

**Table:**

[Ir(C_10_H_15_)(C_11_H_8_N)(N_3_)]	*F*(000) = 1016
*M**_r_* = 523.63	*D*_x_ = 1.934 Mg m^−^^3^
Monoclinic, *P*2_1_/*n*	Mo *K*α radiation, λ = 0.71075 Å
*a* = 15.4821 (18) Å	Cell parameters from 17777 reflections
*b* = 7.3938 (9) Å	θ = 3.0–27.5°
*c* = 15.7137 (18) Å	µ = 7.44 mm^−^^1^
β = 91.477 (4)°	*T* = 193 K
*V* = 1798.2 (4) Å^3^	Needle, orange
*Z* = 4	0.30 × 0.30 × 0.20 mm

### Data collection

**Table:**

Rigaku R-AXIS RAPIDII diffractometer	4022 reflections with *I* > 2σ(*I*)
Detector resolution: 10.000 pixels mm^-1^	*R*_int_ = 0.045
ω scans	θ_max_ = 27.5°, θ_min_ = 3.1°
Absorption correction: numerical (*NUMABS*; Rigaku, 1999)	*h* = −20→20
*T*_min_ = 0.103, *T*_max_ = 0.225	*k* = −9→9
27640 measured reflections	*l* = −20→20
4115 independent reflections	

### Refinement

**Table:**

Refinement on *F*^2^	Primary atom site location: heavy-atom method
Least-squares matrix: full	Secondary atom site location: difference Fourier map
*R*[*F*^2^ > 2σ(*F*^2^)] = 0.017	Hydrogen site location: inferred from neighbouring sites
*wR*(*F*^2^) = 0.042	H-atom parameters constrained
*S* = 1.13	*w* = 1/[σ^2^(*F*_o_^2^) + (0.0078*P*)^2^ + 2.039*P*] where *P* = (*F*_o_^2^ + 2*F*_c_^2^)/3
4115 reflections	(Δ/σ)_max_ = 0.002
240 parameters	Δρ_max_ = 1.61 e Å^−^^3^
0 restraints	Δρ_min_ = −0.65 e Å^−^^3^

### Special details

**Table:**

Geometry. All e.s.d.'s (except the e.s.d. in the dihedral angle between two l.s. planes) are estimated using the full covariance matrix. The cell e.s.d.'s are taken into account individually in the estimation of e.s.d.'s in distances, angles and torsion angles; correlations between e.s.d.'s in cell parameters are only used when they are defined by crystal symmetry. An approximate (isotropic) treatment of cell e.s.d.'s is used for estimating e.s.d.'s involving l.s. planes.

### Fractional atomic coordinates and isotropic or equivalent isotropic displacement parameters (Å^2^)

**Table:**

	*x*	*y*	*z*	*U*_iso_*/*U*_eq_	
Ir1	0.50470 (2)	0.26864 (2)	0.74054 (2)	0.01605 (4)	
N22	0.42229 (13)	0.3666 (3)	0.64468 (14)	0.0241 (4)	
N1	0.55974 (13)	0.5315 (3)	0.73529 (14)	0.0252 (4)	
N2	0.58798 (13)	0.5919 (3)	0.80076 (14)	0.0247 (4)	
N3	0.61722 (17)	0.6587 (3)	0.86156 (16)	0.0384 (6)	
C1	0.51540 (15)	−0.0120 (3)	0.70066 (15)	0.0194 (5)	
C2	0.50048 (15)	−0.0035 (3)	0.79079 (15)	0.0189 (5)	
C3	0.57313 (15)	0.0883 (3)	0.83098 (15)	0.0217 (5)	
C4	0.63164 (15)	0.1339 (3)	0.76593 (16)	0.0229 (5)	
C5	0.59759 (15)	0.0731 (3)	0.68515 (16)	0.0218 (5)	
C6	0.46049 (17)	−0.1057 (3)	0.63532 (16)	0.0261 (5)	
H6A	0.4003	−0.1058	0.6532	0.039*	
H6B	0.4805	−0.2306	0.6289	0.039*	
H6C	0.4645	−0.0424	0.5808	0.039*	
C7	0.42773 (16)	−0.0905 (3)	0.83631 (17)	0.0262 (5)	
H7A	0.4470	−0.2067	0.8600	0.039*	
H7B	0.3791	−0.1104	0.7963	0.039*	
H7C	0.4094	−0.0113	0.8825	0.039*	
C8	0.59008 (19)	0.1038 (4)	0.92487 (17)	0.0331 (6)	
H8A	0.6274	0.2084	0.9366	0.050*	
H8B	0.6187	−0.0064	0.9458	0.050*	
H8C	0.5352	0.1195	0.9537	0.050*	
C9	0.71557 (18)	0.2319 (4)	0.7806 (2)	0.0329 (7)	
H9A	0.7603	0.1457	0.7992	0.049*	
H9B	0.7085	0.3242	0.8246	0.049*	
H9C	0.7327	0.2899	0.7275	0.049*	
C10	0.64306 (17)	0.0740 (4)	0.60188 (17)	0.0314 (6)	
H10A	0.6575	−0.0504	0.5859	0.047*	
H10B	0.6962	0.1454	0.6076	0.047*	
H10C	0.6052	0.1274	0.5577	0.047*	
C11	0.40789 (14)	0.3875 (3)	0.80932 (14)	0.0169 (4)	
C12	0.40386 (17)	0.3927 (3)	0.89675 (16)	0.0266 (5)	
H12	0.4490	0.3388	0.9300	0.032*	
C13	0.33557 (18)	0.4749 (4)	0.93770 (17)	0.0294 (6)	
H13	0.3340	0.4748	0.9981	0.035*	
C14	0.27000 (17)	0.5567 (3)	0.89033 (18)	0.0296 (6)	
H14	0.2231	0.6130	0.9179	0.036*	
C15	0.27330 (16)	0.5559 (3)	0.80278 (18)	0.0267 (5)	
H15	0.2288	0.6129	0.7698	0.032*	
C16	0.34190 (15)	0.4715 (3)	0.76237 (16)	0.0208 (5)	
C17	0.35097 (15)	0.4629 (3)	0.67030 (16)	0.0210 (5)	
C18	0.29488 (16)	0.5441 (3)	0.61069 (17)	0.0265 (5)	
H18	0.2469	0.6122	0.6293	0.032*	
C19	0.30904 (16)	0.5255 (4)	0.52521 (18)	0.0298 (6)	
H19	0.2706	0.5792	0.4845	0.036*	
C20	0.38019 (17)	0.4273 (4)	0.49885 (17)	0.0295 (6)	
H20	0.3907	0.4128	0.4399	0.035*	
C21	0.43519 (16)	0.3516 (3)	0.55904 (16)	0.0251 (5)	
H21	0.4841	0.2862	0.5405	0.030*	

### Atomic displacement parameters (Å^2^)

**Table:**

	*U*^11^	*U*^22^	*U*^33^	*U*^12^	*U*^13^	*U*^23^
Ir1	0.01507 (6)	0.01433 (6)	0.01873 (6)	0.00073 (3)	−0.00018 (4)	0.00032 (3)
N22	0.0226 (10)	0.0210 (10)	0.0287 (11)	−0.0024 (8)	−0.0007 (8)	0.0020 (8)
N1	0.0248 (10)	0.0208 (11)	0.0298 (12)	−0.0029 (8)	−0.0020 (9)	0.0012 (8)
N2	0.0207 (10)	0.0181 (10)	0.0353 (12)	0.0013 (8)	0.0010 (9)	0.0027 (9)
N3	0.0418 (14)	0.0316 (13)	0.0412 (14)	0.0002 (11)	−0.0107 (11)	−0.0059 (11)
C1	0.0219 (11)	0.0142 (11)	0.0220 (12)	0.0027 (9)	0.0001 (9)	0.0005 (8)
C2	0.0198 (11)	0.0151 (11)	0.0218 (12)	0.0022 (9)	−0.0003 (9)	0.0014 (8)
C3	0.0207 (11)	0.0203 (12)	0.0239 (12)	0.0051 (9)	−0.0037 (9)	−0.0010 (9)
C4	0.0175 (11)	0.0162 (11)	0.0348 (14)	0.0037 (9)	−0.0022 (10)	0.0007 (9)
C5	0.0201 (11)	0.0186 (11)	0.0267 (12)	0.0041 (9)	0.0011 (9)	0.0027 (9)
C6	0.0292 (13)	0.0216 (12)	0.0273 (13)	−0.0001 (10)	−0.0050 (10)	−0.0043 (9)
C7	0.0234 (12)	0.0239 (13)	0.0317 (14)	−0.0005 (10)	0.0049 (10)	0.0051 (10)
C8	0.0354 (15)	0.0388 (16)	0.0247 (13)	0.0093 (12)	−0.0077 (11)	−0.0064 (11)
C9	0.0187 (13)	0.0269 (14)	0.053 (2)	−0.0019 (10)	−0.0026 (13)	−0.0053 (11)
C10	0.0271 (13)	0.0379 (15)	0.0295 (14)	0.0073 (11)	0.0077 (11)	0.0069 (11)
C11	0.0166 (10)	0.0131 (10)	0.0210 (11)	−0.0014 (8)	0.0018 (9)	−0.0014 (8)
C12	0.0279 (13)	0.0241 (13)	0.0278 (13)	0.0031 (10)	0.0006 (10)	−0.0011 (10)
C13	0.0338 (14)	0.0270 (13)	0.0279 (13)	−0.0006 (11)	0.0070 (11)	−0.0043 (10)
C14	0.0253 (12)	0.0240 (13)	0.0400 (15)	0.0005 (10)	0.0103 (11)	−0.0068 (11)
C15	0.0192 (11)	0.0205 (12)	0.0403 (15)	0.0014 (10)	0.0011 (10)	−0.0028 (10)
C16	0.0184 (11)	0.0144 (11)	0.0296 (13)	−0.0015 (8)	0.0006 (9)	0.0003 (9)
C17	0.0172 (10)	0.0148 (11)	0.0309 (13)	−0.0041 (9)	−0.0014 (9)	0.0014 (9)
C18	0.0178 (11)	0.0219 (12)	0.0395 (15)	−0.0014 (9)	−0.0021 (10)	0.0074 (10)
C19	0.0233 (12)	0.0302 (14)	0.0354 (15)	−0.0058 (10)	−0.0096 (11)	0.0134 (11)
C20	0.0307 (13)	0.0323 (14)	0.0253 (13)	−0.0062 (11)	−0.0036 (11)	0.0071 (10)
C21	0.0241 (12)	0.0248 (13)	0.0266 (13)	−0.0013 (10)	0.0022 (10)	0.0034 (10)

### Geometric parameters (Å, º)

**Table:**

Ir1—N1	2.125 (2)	C8—H8A	0.9800
Ir1—C11	2.066 (2)	C8—H8B	0.9800
Ir1—N22	2.079 (2)	C8—H8C	0.9800
Ir1—C1	2.175 (2)	C9—H9A	0.9800
Ir1—C2	2.163 (2)	C9—H9B	0.9800
Ir1—C3	2.201 (2)	C9—H9C	0.9800
Ir1—C4	2.230 (2)	C10—H10A	0.9800
Ir1—C5	2.232 (2)	C10—H10B	0.9800
N1—N2	1.194 (3)	C10—H10C	0.9800
N2—N3	1.157 (3)	C11—C12	1.377 (3)
N22—C17	1.383 (3)	C11—C16	1.391 (3)
N22—C21	1.370 (3)	C12—C13	1.391 (4)
C1—C2	1.442 (3)	C12—H12	0.9500
C1—C5	1.446 (3)	C13—C14	1.383 (4)
C1—C6	1.487 (3)	C13—H13	0.9500
C2—C3	1.445 (3)	C14—C15	1.378 (4)
C2—C7	1.495 (3)	C14—H14	0.9500
C3—C4	1.424 (3)	C15—C16	1.398 (3)
C3—C8	1.496 (3)	C15—H15	0.9500
C4—C5	1.434 (3)	C16—C17	1.459 (3)
C4—C9	1.500 (3)	C17—C18	1.396 (3)
C5—C10	1.502 (3)	C18—C19	1.373 (4)
C6—H6A	0.9800	C18—H18	0.9500
C6—H6B	0.9800	C19—C20	1.391 (4)
C6—H6C	0.9800	C19—H19	0.9500
C7—H7A	0.9800	C20—C21	1.376 (4)
C7—H7B	0.9800	C20—H20	0.9500
C7—H7C	0.9800	C21—H21	0.9500
			
C11—Ir1—N22	77.94 (9)	C1—C6—H6A	109.5
C11—Ir1—N1	85.90 (8)	C1—C6—H6B	109.5
N22—Ir1—N1	83.83 (8)	H6A—C6—H6B	109.5
C11—Ir1—C1	128.17 (9)	C1—C6—H6C	109.5
N22—Ir1—C1	99.98 (9)	H6A—C6—H6C	109.5
N1—Ir1—C1	145.88 (9)	H6B—C6—H6C	109.5
C11—Ir1—C2	100.10 (9)	C2—C7—H7A	109.5
N22—Ir1—C2	124.38 (8)	C2—C7—H7B	109.5
N1—Ir1—C2	151.76 (9)	H7A—C7—H7B	109.5
C11—Ir1—C3	105.28 (9)	C2—C7—H7C	109.5
N22—Ir1—C3	162.78 (8)	H7A—C7—H7C	109.5
N1—Ir1—C3	113.11 (9)	H7B—C7—H7C	109.5
C11—Ir1—C4	138.08 (9)	C3—C8—H8A	109.5
N22—Ir1—C4	143.78 (9)	C3—C8—H8B	109.5
N1—Ir1—C4	93.62 (8)	H8A—C8—H8B	109.5
C11—Ir1—C5	164.32 (8)	C3—C8—H8C	109.5
N22—Ir1—C5	109.43 (9)	H8A—C8—H8C	109.5
N1—Ir1—C5	108.32 (9)	H8B—C8—H8C	109.5
C1—Ir1—C2	38.84 (9)	C4—C9—H9A	109.5
C1—Ir1—C3	64.47 (9)	C4—C9—H9B	109.5
C1—Ir1—C4	63.39 (9)	H9A—C9—H9B	109.5
C1—Ir1—C5	38.27 (9)	C4—C9—H9C	109.5
C2—Ir1—C3	38.66 (9)	H9A—C9—H9C	109.5
C2—Ir1—C4	63.51 (9)	H9B—C9—H9C	109.5
C2—Ir1—C5	64.25 (9)	C5—C10—H10A	109.5
C3—Ir1—C4	37.47 (9)	C5—C10—H10B	109.5
C3—Ir1—C5	63.57 (9)	H10A—C10—H10B	109.5
C4—Ir1—C5	37.50 (9)	C5—C10—H10C	109.5
N2—N1—Ir1	116.55 (17)	H10A—C10—H10C	109.5
N1—N2—N3	176.0 (3)	H10B—C10—H10C	109.5
C21—N22—Ir1	125.55 (17)	C12—C11—C16	117.7 (2)
C17—N22—Ir1	116.63 (16)	C12—C11—Ir1	125.87 (18)
C21—N22—C17	117.7 (2)	C16—C11—Ir1	116.43 (17)
C2—C1—C5	108.1 (2)	C11—C12—C13	121.9 (2)
C2—C1—C6	126.5 (2)	C11—C12—H12	119.1
C5—C1—C6	125.2 (2)	C13—C12—H12	119.1
C2—C1—Ir1	70.14 (12)	C14—C13—C12	119.9 (2)
C5—C1—Ir1	73.00 (13)	C14—C13—H13	120.1
C6—C1—Ir1	126.65 (17)	C12—C13—H13	120.1
C1—C2—C3	107.9 (2)	C15—C14—C13	119.3 (2)
C1—C2—C7	126.4 (2)	C15—C14—H14	120.3
C3—C2—C7	125.5 (2)	C13—C14—H14	120.3
C1—C2—Ir1	71.03 (12)	C14—C15—C16	120.2 (2)
C3—C2—Ir1	72.09 (13)	C14—C15—H15	119.9
C7—C2—Ir1	127.23 (16)	C16—C15—H15	119.9
C4—C3—C2	107.5 (2)	C11—C16—C15	121.0 (2)
C4—C3—C8	126.2 (2)	C11—C16—C17	114.7 (2)
C2—C3—C8	125.6 (2)	C15—C16—C17	124.3 (2)
C4—C3—Ir1	72.38 (14)	N22—C17—C18	120.9 (2)
C2—C3—Ir1	69.26 (13)	N22—C17—C16	114.1 (2)
C8—C3—Ir1	131.27 (18)	C18—C17—C16	125.0 (2)
C3—C4—C5	109.6 (2)	C19—C18—C17	120.1 (2)
C3—C4—C9	124.7 (2)	C19—C18—H18	120.0
C5—C4—C9	125.8 (2)	C17—C18—H18	120.0
C3—C4—Ir1	70.14 (13)	C18—C19—C20	119.4 (2)
C5—C4—Ir1	71.31 (13)	C18—C19—H19	120.3
C9—C4—Ir1	124.56 (17)	C20—C19—H19	120.3
C4—C5—C1	107.0 (2)	C21—C20—C19	119.2 (3)
C4—C5—C10	126.9 (2)	C21—C20—H20	120.4
C1—C5—C10	125.7 (2)	C19—C20—H20	120.4
C4—C5—Ir1	71.19 (13)	N22—C21—C20	122.6 (2)
C1—C5—Ir1	68.73 (13)	N22—C21—H21	118.7
C10—C5—Ir1	131.16 (17)	C20—C21—H21	118.7
			
C5—C1—C2—C3	0.5 (2)	C6—C1—C5—C4	−175.5 (2)
C6—C1—C2—C3	175.4 (2)	Ir1—C1—C5—C4	61.27 (16)
Ir1—C1—C2—C3	−63.08 (16)	C2—C1—C5—C10	172.1 (2)
C5—C1—C2—C7	−173.7 (2)	C6—C1—C5—C10	−2.9 (4)
C6—C1—C2—C7	1.3 (4)	Ir1—C1—C5—C10	−126.2 (2)
Ir1—C1—C2—C7	122.8 (2)	C2—C1—C5—Ir1	−61.72 (15)
C5—C1—C2—Ir1	63.57 (15)	C6—C1—C5—Ir1	123.2 (2)
C6—C1—C2—Ir1	−121.5 (2)	C16—C11—C12—C13	1.5 (4)
C1—C2—C3—C4	−0.3 (3)	Ir1—C11—C12—C13	−179.50 (19)
C7—C2—C3—C4	173.9 (2)	C11—C12—C13—C14	−1.0 (4)
Ir1—C2—C3—C4	−62.72 (16)	C12—C13—C14—C15	−0.1 (4)
C1—C2—C3—C8	−171.0 (2)	C13—C14—C15—C16	0.6 (4)
C7—C2—C3—C8	3.2 (4)	C12—C11—C16—C15	−0.9 (3)
Ir1—C2—C3—C8	126.6 (2)	Ir1—C11—C16—C15	179.99 (18)
C1—C2—C3—Ir1	62.40 (15)	C12—C11—C16—C17	179.1 (2)
C7—C2—C3—Ir1	−123.4 (2)	Ir1—C11—C16—C17	0.0 (3)
C2—C3—C4—C5	0.0 (3)	C14—C15—C16—C11	−0.1 (4)
C8—C3—C4—C5	170.6 (2)	C14—C15—C16—C17	179.8 (2)
Ir1—C3—C4—C5	−60.66 (16)	C21—N22—C17—C18	−0.9 (3)
C2—C3—C4—C9	179.6 (2)	Ir1—N22—C17—C18	175.84 (17)
C8—C3—C4—C9	−9.8 (4)	C21—N22—C17—C16	179.7 (2)
Ir1—C3—C4—C9	118.9 (2)	Ir1—N22—C17—C16	−3.6 (3)
C2—C3—C4—Ir1	60.70 (16)	C11—C16—C17—N22	2.3 (3)
C8—C3—C4—Ir1	−128.7 (3)	C15—C16—C17—N22	−177.7 (2)
C3—C4—C5—C1	0.3 (3)	C11—C16—C17—C18	−177.1 (2)
C9—C4—C5—C1	−179.3 (2)	C15—C16—C17—C18	3.0 (4)
Ir1—C4—C5—C1	−59.68 (15)	N22—C17—C18—C19	1.4 (4)
C3—C4—C5—C10	−172.2 (2)	C16—C17—C18—C19	−179.2 (2)
C9—C4—C5—C10	8.2 (4)	C17—C18—C19—C20	−0.8 (4)
Ir1—C4—C5—C10	127.9 (2)	C18—C19—C20—C21	−0.3 (4)
C3—C4—C5—Ir1	59.94 (16)	C17—N22—C21—C20	−0.3 (4)
C9—C4—C5—Ir1	−119.6 (2)	Ir1—N22—C21—C20	−176.68 (19)
C2—C1—C5—C4	−0.5 (2)	C19—C20—C21—N22	0.9 (4)
